# Effectiveness Studies in Health Promotion: A Review of the Methodological Quality of Studies Reporting Significant Effects on Physical Activity in Working Age Adults

**DOI:** 10.3390/ijerph16050813

**Published:** 2019-03-06

**Authors:** Kevin Rudolf, Lea A. L. Dejonghe, Ingo Froböse, Florian Lammer, Lisa-Marie Rückel, Jessica Tetz, Andrea Schaller

**Affiliations:** 1 Institute of Movement Therapy and movement-oriented Prevention and Rehabilitation, German Sport University Cologne, Am Sportpark Muengersdorf 6, 50933 Cologne, Germany; lea.dejonghe@web.de (L.A.L.D.); froboese@dshs-koeln.de (I.F.); florian.lammer@t-online.de (F.L.); lissy.rueckel@gmx.de (L.-M.R.); jessicatetz@t-online.de (J.T.); a.schaller@dshs-koeln.de (A.S.); 2 Center for Health and Physical Activity, German Sport University Cologne, Am Sportpark Muengersdorf 6, 50933 Cologne, Germany; 3 IST-University of Applied Sciences, Erkrather Straße 220 a-c, 40233 Duesseldorf, Germany

**Keywords:** evidence development, physical activity promotion, methodology, assessment, study design, reporting guidelines

## Abstract

The methodology of intervention studies on physical activity (PA) promotion is of great importance regarding evidence development in complex interventions. The aim of this review was to provide an overview of the methodological quality of those studies which reported statistically significant effects of interventions promoting PA. PUBMED was searched for reviews on PA promotion to identify studies reporting effective interventions with participants of working age (16–67 years). Selected reviews were screened and data from primary studies with effective interventions were extracted to assess methodological quality. Forty-six reviews with 600 primary studies were identified, of which 33 met the inclusion criteria. Twenty-one studies were conducted as randomized controlled trials, 13 included an intervention control group, 25 measured PA by questionnaire, and 13 included objective measurements. Information on used statistics was often scarce, and long-term follow-up measurements were frequently missing. The overall methodological quality was moderate for randomized studies and low for non-randomized studies; information on methods and results was often lacking. To overcome these methodological issues, standardized guidelines for reporting study results should be considered, not only when publishing results but also when designing studies. This review provides a solid foundation for the development of practical advice for planning application-oriented studies in PA promotion.

## 1. Introduction

Physical activity (PA) is a widely accepted cornerstone of a healthy lifestyle and a favored target of public health efforts [1]. Nevertheless, promoting physical activity remains a challenge due to the complexity of the interventions and the various influencing factors such as personal, social, and environmental conditions [2,3,4]. From a practice-oriented perspective, the question of the intervention components which are associated with increased effectiveness in promoting PA, e.g., setting, delivery mode, study population or delivery provider, are of high relevance for maximizing the effectiveness of PA promotion programs [5,6]. However, there is considerable heterogeneity in promoting PA regarding settings and content, as well as the delivery mode and means of access [7,8]. In this context, several systematic reviews have already tried to identify components associated with the effectiveness of PA promotion, although most of these reviews have shown inconclusive results [6,8,9,10]. Overall, the reviews could not prove a clear relationship between effectiveness and the intervention components. Neither the intervention setting [9,10] nor the delivery mode [5,8,9,10] showed homogenous results.

While the question of the effectiveness of specific intervention components is of great interest for practitioners, the methodology of intervention studies on PA promotion is of great importance regarding evidence development in complex interventions. To provide the best internal validity possible, interventions on promoting PA should refer to the standards of evidence-based medicine [11,12] including the use of the best available methods as well as the precise reporting of methods and results. While randomized controlled trials (RCTs) are considered the gold standard for high internal validity [13,14], the feasibility of RCTs in PA promotion is widely debated [15,16]. Among other things, the practicability of RCTs in the field of multifaceted and complex interventions as well as the accompanying costs are questioned [16]. With regard to reporting, several guidelines have emerged over the years. According to the CONSORT checklist for the transparent reporting of trials [14], the trial should report, inter alia, the description of the trial design, the sample size calculation as well as the completely defined, pre-specified primary and secondary outcome measures, including how they were assessed [14]. 

The aim of the present review was to assess the methodological quality of studies reporting effective interventions in PA promotion. Due to the high number of studies investigating interventions in PA promotion, the present review focused on studies reporting effective interventions since those are the ones being read and cited most often [17,18]. Moreover, previous research has shown that methodological quality influences conclusions of effectiveness in such a way that poorer study quality increases the likelihood of reporting statistically significant effects [19,20].

## 2. Materials and Methods 

This review was conducted following the international guidelines established by PRISMA (preferred reporting items for systematic reviews and meta-analyses) [21]. Since this review was based on a review of the literature, no ethical approval was required.

### 2.1. Search Strategy and Data Source

This review focused on articles published between January 2007 and August 2016 in English or German. To include studies which had already been cited elsewhere, two researchers (JT, FL) independently searched the database PUBMED for reviews. The following keywords (including medical subject headings) were used and combined by the Boolean operator “AND”: physical activity, promotion, and intervention. The tags “child” and “school” were excluded by the Boolean operator “NOT” to directly reduce the number of results. Truncations (child*, school*) were used. Subsequently, the reviews identified were checked for primary studies reporting effectiveness in PA promotion. In an additional manual search, reviews of reviews and review protocols were checked for further reviews which fit the inclusion criteria and were not found in the PUBMED search. 

### 2.2. Study Selection

In the first search, the article type filter was set to review. In a two-phase screening process, first, the reviews’ titles and abstracts were independently screened for eligibility by two researchers (JT, FL). In the second phase, the full texts of relevant reviews were acquired and assessed against the inclusion criteria, which were 1) reviews or meta-analyses focusing on 2) the effectiveness of PA interventions among 3) participants of working age (16–67 years). Reviews were excluded if 1) PA was not a primary outcome, and 2) they only focused on sedentary behavior (e.g., sitting time), fitness (e.g., maximal oxygen consumption), or vital parameters (e.g., blood pressure) (see Table 1). If a selected review did not report information about the participants’ age range or the primary outcome, the review was provisionally included, and its primary studies were later checked separately for these inclusion criteria. If none of the primary studies complied with the inclusion criteria, the whole review was excluded subsequently.

Based on the results of the review search, the primary studies of the reviews reporting an effective PA intervention were included in the second search. After excluding duplicates, two researchers (JT, FL) screened the titles and abstracts and independently checked the following inclusion criteria: 1) published 2007 or later, 2) primary outcome PA (frequency, duration and/or intensity), and 3) the authors reported a statistically significant effectiveness of the intervention for the PA outcome. Exclusion criteria for the primary studies in the second search were the same as described for the first search. The full texts were assessed by the researchers if the relevant information for inclusion could not be identified by the abstract screening process. Disagreements regarding inclusion were resolved through discussion involving the two researchers checking for eligibility (JT, FL) and an additional researcher (KR). Full (100%) consensus was achieved. All the studies that were excluded during the screening processes were recorded, along with the reasons for exclusion.

### 2.3. Data Collection

The data extraction was performed by one researcher (LMR) and cross-checked by a second researcher (KR), with reference to the full text of the article. Following the CONSORT checklist [14], data on ten predefined study components were extracted and summarized in a data extraction template:

1. Study design;
2. Control group (CG) condition (intervention CG (we define an intervention CG as a group that receives any form of intervention, including usual care and placebos, and is called a CG by the authors of the primary study), non-intervention CG (we define a non-intervention CG as a group which is instructed to continue their lifestyle.) or no CG);
3. Sample size (number of participants analyzed in primary outcome results);
4. Operationalization of PA (subjective and/or objective measurements, observed time frame);
5. Reporting of sample size calculation and achievement of desired number of cases (yes/no);
6. Reporting of intention-to-treat (ITT) analyses (we define ITT analyses as analyses of study participants according to the original group allocation, regardless of non-compliance or inconsistency with the study protocol. We did not differentiate between the different methods of handling missing outcome data [22]) (yes/no);
7. Checking for baseline group differences (yes/no);
8. Reporting of drop-out analyses (yes/no);
9. Reporting of standardized effect sizes of the results (e.g., Cohen’s d);
10. Follow-up measurements (after the measurement at the end of the intervention).

In addition, the Delphi list [23] was used to assess the methodological quality of the studies included. This list is commonly used in systematic reviews [24,25] and has a comparatively greater validity of evidence than other standardized quality checklists [26]. Two researchers (LMR, KR) independently rated the studies’ quality by assigning a value of 0 or 1 for the 9 items of the Delphi list (1 point = “yes”; 0 point = “no” or “don’t know/not reported”). An interrater reliability analysis for individual Delphi scores was performed using the Kappa statistic.

## 3. Results

**Figure 1:**
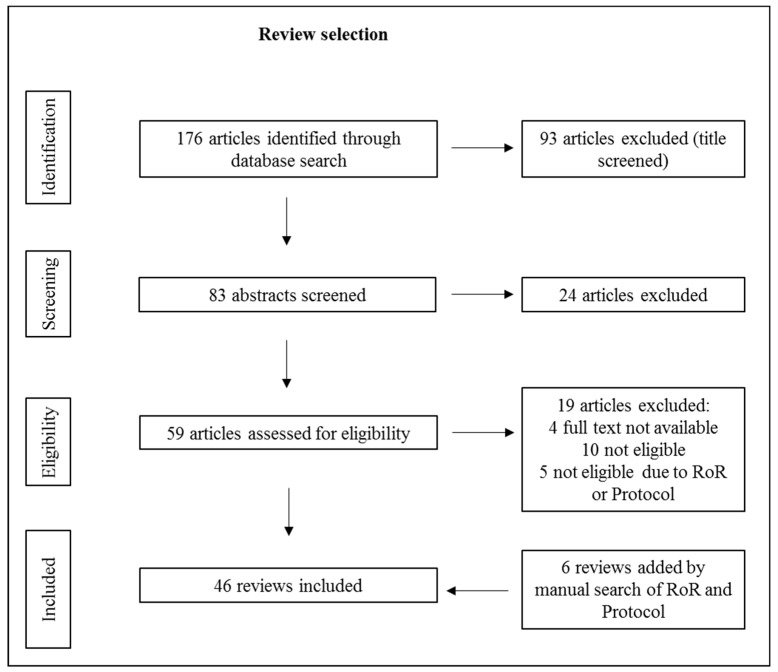
Flow chart of the review selection process (step 1). Since not all included reviews provided suitable primary studies, we provide a Supplementary appendix with all reviews from which we included primary studies.

**Figure 2:**
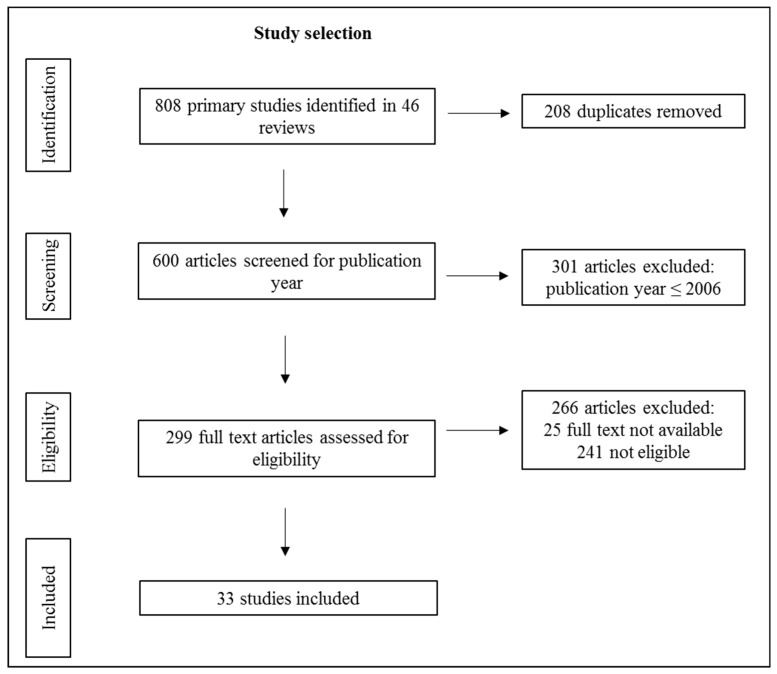
Flow chart of the study selection process (step 2).

The combination of the keywords resulted in a total of 176 reviews whose titles and abstracts were screened for eligibility. In the next step, the full texts of the remaining 59 reviews were checked. A total of 19 reviews were excluded because of exclusion criteria being found in the full text. Five of these were provisionally excluded because they were reviews of reviews (RoR) or review protocols, which were later used for a manual search. Six reviews were added by manual search. Therefore, 46 reviews were included, resulting in 808 primary studies about PA interventions. After removing duplicates and checking for eligibility, 775 studies were excluded. As a result, a total of 33 studies were included in the present review. The flow chart diagrams (see Figure 1 and Figure 2) give an overview of the literature search process.

Table 2 shows the results of the data extraction process. The individual Delphi scores for the studies included a range between 0 and 7 points, with an average of 5.1 out of 9 possible points for randomized studies and 1.3 points for non-randomized studies (see Table 2). The interrater reliability analysis showed a very good [25] interrater agreement with Kappa = 0.93 (*p* < 0.001) for individual Delphi scores. No study attained the maximum score of nine points. Four studies included the blinding of the outcome accessors, patients, and/or an intervention provider. No other study reported or used any kind of blinding procedure. 

The biggest similarities between the studies included were in the study design. The majority of the studies (*n* = 27; 81.8%) were conducted as randomized trials, 21 thereof (63.6%) as RCT. Correspondingly, most studies (*n* = 26; 78.8%) had some kind of control group condition. While 13 (39.4%) had an intervention control group, 13 (39.4%) used a non-intervention control group and seven (21.2%) had no control group at all. Of those without a control group, four studies had only one intervention group, one had two intervention groups and two had three such groups.

Moreover, almost all (*n* = 26) of the studies with more than one group (*n* = 29) presented results for baseline group differences. 

The sample sizes of the studies included ranged from 25 to 1239 research participants, with most studies (*n* = 19, 57.6%) having more than 100 participants in total.

Another similarity between the included studies was the absence of long-term follow-up measurements. Only four (12.1%) studies presented follow-up results based on an additional measurement after the measurement at the end of the intervention period. 

The biggest differences between the studies included the operationalization of PA. While the time frame predominantly focused on a typical week or the last seven days (*n* = 23, 69.7%), the measures were varied. More than half of the studies (*n* = 20; 60.6%) used subjective measures, while about a quarter (*n* = 8; 24.2%) reported objective measurements and five studies (15.2%) a combination of both. The most commonly used subjective measure was the International Physical Activity Questionnaire [59] (*n* = 9, 27.3%). Pedometers (*n* = 7, 21.2%) and accelerometers (*n* = 6, 18.2%) were used almost equally as objective measures. Of those using objective measures, six studies instructed participants to manually record their daily/weekly data in a step log.

Further differences are related to the reporting of the statistics used and results. Less than a third (*n* = 10; 30.3%) of the studies conducted an a priori sample size calculation, 14 (42.4%) studies reported ITT analyses and less than a third (*n* = 9; 27.3%) included a standardized effect size in the description of the individual results. Moreover, 11 (33.3%) studies performed a drop-out analysis.

## 4. Discussion

The aim of the present review was to assess the methodological quality of intervention studies which effectively promoted PA. In total, we summarized data from 33 effectiveness studies reporting effective interventions in PA promotion, most of which were conducted as RCTs with either intervention or non-intervention control groups, moderate Delphi scores and usually more than 100 participants overall. 

In terms of data collection, questionnaires were used more frequently than accelerometers and pedometers. Follow-up data after the measurement at the end of the intervention period were usually not collected or reported. The biggest differences between the studies were found in the reporting of the statistics used. While most studies checked the baseline data for statistically significant group differences, ITT and dropout analyses as well as sample and effect size calculations were reported considerably less often.

Overall, the methodological quality of the 33 studies which effectively promoted PA was moderate. While most studies applied some of the standard methods of securing high quality, such as the use of randomized controlled designs [13,14], there is still a lot of room for improvement regarding the reporting of methods (e.g., sample size calculation) and results (e.g., standardized effect sizes). Although reporting guidelines such as CONSORT [14] have existed for several years and many journals refer to the CONSORT statement in their “instructions to authors” section [60], the information within the studies published before and after the release of the CONSORT statement remains heterogeneous. For example, only a third of all studies included in our review reported a priori sample size calculations, although the benefits of an optimal sample size is well known: too-small sample sizes are more prone to bias [61,62], whereas a large number of participants consumes more resources and facilitates the detection of statistically significant changes which are not necessarily clinically relevant results [62,63], not to mention the ethical issues arising from exposing large numbers of participants to possibly non-effective interventions [62,64].

A pleasing result of the current review was the prevalence of RCTs. The promotion of PA usually takes place in complex settings [65,66], where it is hard to administer RCT implementation. Nevertheless, the results of the present review show that more than 80% of the identified studies managed to include some kind of randomization procedure, showing that an approximation to the standards of evidence-based medicine [11,12] is possible and that statistically significant results can be obtained under these complex conditions. Furthermore, four studies even managed to apply blinding procedures. This is a positive sign for evidence development and should be an incentive for researchers to use RCTs and, thus, secure high quality in future studies [13,14].

In line with the prevalent use of RCTs, the majority of the studies included control groups or used various intervention groups to examine the effectiveness of the individual interventions. However, a more frequent use of intervention control groups is desirable, since non-intervention control groups cannot rule out a possible placebo-effect of the interventions [67]. Moreover, intervention control groups would add knowledge through the comparison of different kinds of interventions and, hence, facilitate the search for the most effective interventions [67]. 

An even further strengthening of the research would be the more frequent reporting of standardized effect sizes, which would allow quantitative comparison between different treatments [63,67,68] and could also be used for sample size calculations in advance of a study [63].

A striking feature of our results is the frequent use of questionnaires as the instrument of choice for the operationalization of PA. It stands to reason that subjective measurements increase the probability of a study to be reported as effective, but it is noticeable that more than 75% of the studies included a subjective measurement for the primary outcome, whereas only five studies used an additional objective measure. Obviously, data collection via questionnaires is much cheaper and easier to manage than most objective assessments [69,70], especially in large sample sizes. However, since both subjective and objective measures have individual strengths and weaknesses [71] and the respective data can differ widely [69], a combination of both is advisable to adjust for the individual weaknesses and to obtain a holistic view of individuals’ PA levels [72]. A positive aspect of additionally using accelerometers or pedometers is its objectivity, or rather that it is independent of the participants’ ability to accurately recollect the duration and intensity of their PA, which is often the subject of misperception [69,73,74]. The often-used method of letting participants write down their objectively measured data in a daily log (and then only evaluating this log data), however, reduces the objectivity of the data collection process since it brings back social desirability and the possibility of data bias. A possible solution to this problem could be the use of devices with sufficient memory capacity, so that the need to record data by hand becomes obsolete. 

The present review shows that the application of standardized reporting guidelines needs to become more established in the field of PA promotion. Moreover, these guidelines should not only be used when publications are being written, but also should be considered when studies are planned, to make sure that all of the necessary information will be available when the results are published. 

### Study Limitations and Strengths 

To our knowledge, this review is the first to address the methodological quality of studies effectively promoting PA. Our review does not claim to provide a full overview of the methodology used in the studies. Instead, it is limited to those studies reporting successful interventions, since effective studies are usually those who attract more attention by being cited more often [17,18]. In line with that, we included studies that had already been cited in other reviews. The aim was to consider their specific methodological structure to provide implications for future studies and to sensitize readers to methodological issues when interpreting the respective results. Nonetheless, studies showing non-effective results should have the same methodological quality as studies with positive findings. Methodologically poor quality studies can not only lead to the overrating of false positive results but also to the erroneous rejection of interventions that would have shown effectivity if evaluated better. Moreover, the need for good methodology is not only restricted to effectiveness studies but also applies to every other kind of study and research question, respectively.

Due to the large number of studies investigating PA promotion, we restricted our literature search to one database and studies which investigated participants of working age (16–67 years). Moreover, the decision to limit the searches to articles published between 2007 and 2016 was a pragmatic one to reduce the number of included studies to a manageable amount. Wider inclusion criteria may have resulted in a larger number of studies being included and a different occurrence of the selected methodological characteristics. Nevertheless, our study could demonstrate that the methodological quality of studies leaves a lot of room for improvement and that attention should be paid to study quality when summarizing study results.

The selection of the ten methodological study components for this study, in addition to the standardized Delphi list, is another strength of this review. We included different components such as the operationalization of PA, which are not covered by the Delphi list, to provide an extensive view of each study’s methodology, with a special focus on PA research. However, it must be mentioned that the Delphi list was originally created for RCTs [23]. Although almost two-thirds of the identified studies were RCTs, the Delphi list may be regarded as a suboptimal choice for the quality assessment. Although other quality assessment methods which are able to deal with non-randomized trials exist (e.g., the EPHPP Quality Assessment tool [75] and ROBINS-I [76]), we chose the Delphi list because of its psychometric values [26] and its frequent use in reviews [24,25]. Moreover, along with other quality assessment tools for RCTs, the Delphi list is often used as the basis for developing further scales [26]. For these reasons and because we did not want to combine two different assessment tools, we decided to also apply the Delphi list to non-RCTs.

In addition to that, some of the reviews from which we extracted primary studies used quality assessment tools as well. However, due to the diverse instruments used in those reviews as well as missing quality appraisal for some of the primary studies, we decided to not include the quality assessment of the other reviews in our results.

## 5. Conclusions

Methodological weaknesses may increase the probability of bias and unknown sources of error affecting study results. From a scientific point of view, the broad implementation of RCTs in the investigated intervention studies is pleasing, but weaknesses in the reporting of methods and results could still be identified. The challenge remains of overcoming the weaknesses identified and increasing the quality and explanatory power of study results; especially, the reporting of statistics, the combination of measurements as well as the use of long-term follow up measurements need to be improved. A more frequent adherence to guidelines for publishing study results is advisable. In addition to the existing guidelines for the publication of study results, guidelines for designing and conducting application-oriented studies on promoting PA are needed. The present review provides a first step for the development of these guidelines. 

## Figures and Tables

**Table 1:** Eligibility criteria for the literature search. PA: physical activity.

Selection Process	Inclusion Criteria	Exclusion Criteria
First phase (review selection)	Article type: reviews or meta-analyses Focus: effectiveness of PA interventions Participants: working age (16–67 years)	Primary outcome: not PA Focus: only sedentary behavior (e.g., sitting time), fitness (e.g., maximal oxygen consumption), or vital parameters (e.g., blood pressure)
Second phase (study selection)	Published: 2007 or later Primary outcome: PA (frequency, duration and/or intensity) Authors reported statistically significant effectiveness of the intervention	Same as the exclusion criteria of the first search

**Table 2:** Methodological components of effective PA interventions.

Study	Delphi List	Materials and Methods					Results		
Author, Year	Score [0–9]	Study Design	Group Conditions (Sample Size)	Operationalization of PA (Instrument; Time Frame)		Statistics: ▪ Sample Size Calculation (SSC) ▪ ITT Analysis (ITT) ▪ Check for Baseline Differences (CBD) ▪ Drop-Out Analysis (DOA)	Sample Size Achieved	Effect Size	Follow-Up after end of Intervention (Time; Significant Difference from Baseline)
				Subjective	Objective				
Allen et al., 2008 [27]	5	RCT	IG (*n* = 21) ICG (*n* = 25)	-	✓ (Accelerometer, 7 days)	▪ SSC - ▪ ITT - ▪ CBD ✓ ▪ DOA -	-	✓	-
Baker et al., 2008 [28]	6	RCT	IG (*n* = 39) Non-ICG (*n* = 40)	✓ (IPAQ, 7 days)	✓ (Pedometer, 7 days)	▪ SSC ✓ ▪ ITT ✓ ▪ CBD ✓ ▪ DOA ✓	✓	✓	-
Carr et al., 2008 [29]	5	RCT	IG (*n* = 14) Non-ICG (*n* = 18)	-	✓ (Pedometer, 7 days^a^)	▪ SSC ✓ ▪ ITT - ▪ CBD ✓ ▪ DOA ✓	-	✓	-
Cheung, Chow, & Parfitt, 2008 [30]	3	RCT	IG (*n* = 38) Non-ICG (*n* = 14)	-	✓ (Pedometer, 5 days^a^)	▪ SSC - ▪ ITT - ▪ CBD ✓ ▪ DOA -	-	-	-
Conroy et al., 2011 [31]	4	Randomized clinical trial: secondary analysis	IG1 (*n* = 61) IG2 (*n* = 64) IG3 (*n* = 64) No CG	✓ (Modifiable Activity Questionnaire, 6 months)	-	▪ SSC - ▪ ITT- ▪ CBD ✓ ▪ DOA -	-	-	-
Dinger et al., 2007 [32]	5	Randomized clinical trial	IG1 (*n* = 24) IG2 (*n* = 32) No CG	✓ (Selection of IPAQ short version items, 7 days)	-	▪ SSC - ▪ ITT - ▪ CBD ✓ ▪ DOA -	-	✓	-
Dirige et al., 2013 [33]	4	RCT	IG (*n* = 255) ICG (*n* = 273)	✓ (Godin Shephard Physical Activity Survey, 7 days)	-	▪ SSC - ▪ ITT - ▪ CBD ✓ ▪ DOA -	-	✓	-
Dunton & Robertson, 2008 [34]	6	RCT	IG (*n* = 85) Non-ICG (*n* = 71)	✓ (Standardized activity inventory format, 2 weeks)	-	▪ SSC ✓ ▪ ITT ✓ ▪ CBD ✓ ▪ DOA -	-	-	-
Ferney et al., 2009 [35]	7	RCT	IG (*n* = 52) ICG (*n* = 54)	✓ (Active Australian Questionnaire, 7 days; Self-reported neighborhood walking, 7 days)	-	▪ SSC - ▪ ITT ✓ ▪ CBD ✓ ▪ DOA -	-	-	-
Gilson, et al., 2007 [36]	3	RCT	IG1 (*n* = 21) IG2 (*n* = 21) Non-ICG (*n* = 22)	-	✓ (Pedometer, 5 days)	▪ SSC - ▪ ITT - ▪ CBD ✓ ▪ DOA -	-	✓	-
Hemmingsson et al., 2008 [37]	3	Randomized clinical trial	IG (*n* = 22) ICG (*n* = 20)	-	✓ (Pedometer, 7 days^a^)	▪ SSC ✓ ▪ ITT - ▪ CBD - ▪ DOA -	✓	-	-
Hooker et al., 2011 [38]	2	Quasi experimental pre-post design	IG (*n* = 25) No CG	✓ (CHAMPS, 7 days)	-	▪ SSC - ▪ ITT - ▪ CBD - ▪ DOA -	-	-	-
Hurling et al., 2007 [39]	6	RCT	IG (*n* = 47) Non-ICG (*n* = 30)	✓ (IPAQ, 7 days)	✓ (Accelerometer, 12 weeks)	▪ SSC - ▪ ITT ✓ ▪ CBD ✓ ▪ DOA -	-	-	-
Katz et al., 2008 [40]	5	Controlled educational trial	IG (*n* = 185) ICG (*n* = 117)	✓ (Yale Physical Activity Survey, 7 days)	-	▪ SCC - ▪ ITT - ▪ CBD ✓ ▪ DOA -	-	-	✓ (6 months, significant)
Kwak et al., 2007 [41]	1	Cohort study	IG (approx. *n* = 950) No CG	-	✓ (Count of staircase use, 3 weeks)	▪ SSC - ▪ ITT - ▪ CBD - ▪ DOA -	-	-	✓ (1 week, non-significant)
Lane et al., 2010 [42]	2	RCT	IG (*n* = 55) ICG (*n* = 57)	✓ (Subjective questions, 7 days)	-	▪ SSC - ▪ ITT - ▪ CBD ✓ ▪ DOA ✓	-	-	-
Liebreich et al., 2009 [43]	6	RCT	IG (*n* = 23) ICG (*n* = 24)	✓ (GLTEQ modified, 1 month)	-	▪ SSC - ▪ ITT ✓ ▪ CBD ✓ ▪ DOA ✓	-	✓	-
Marcus et al., 2007 [44]	6	RCT	IG1 (*n* = 80) IG2 (*n* = 81) ICG (*n* = 78)	✓ (7-day PAR)	-	▪ SSC - ▪ ITT ✓ ▪ CBD ✓ ▪ DOA -	-	-	-
Merom et al., 2007 [45]	7	RCT	IG1 (*n* = 123) IG2 (*n* = 123) Non-ICG (*n* = 123)	✓ (Active Australian Questionnaire, 7 days; College Alumni Questionnaire, 3 months)	-	▪ SSC ✓ ▪ ITT ✓ ▪ CBD ✓ ▪ DOA ✓	✓	-	-
Migneault et al., 2012 [46]	4	RCT	IG (*n* = 169) ICG (*n* = 168)	✓ (7-day PAR)	-	▪ SSC ✓ ▪ ITT ✓ ▪ CBD ✓ ▪ DOA ✓	✓	-	-
Oenema et al., 2008 [47]	6	RCT	IG (*n* = 462) ^b^ Non-ICG (*n* = 504)	✓ (Short-form IPAQ, 7 days)	-	▪ SSC ✓ ▪ ITT ✓ ▪ CBD ✓ ▪ DOA ✓	✓	-	-
Opdenacker et al., 2008 [48]	5	RCT	IG (*n* = 68) Non-ICG (*n* = 60)	✓ (IPAQ, 7 days)	✓ (Accelerometer, 5 days ^a^)	▪ SSC - ▪ ITT ✓ ▪ CBD ✓ ▪ DOA ✓	-	-	-
Pekmezi et al., 2010 [49]	4	RCT	IG1 (*n* = 11) IG2 (*n* = 15) ICG (*n* = 12)	✓ (7-day PAR)	-	▪ SSC - ▪ ITT - ▪ CBD - ▪ DOA -	-	-	-
Prestwich, Perugini, & Hurling, 2010 [50]	7	RCT	IG1 (*n* = 40) IG2 (*n* = 48) ICG (*n* = 46)	✓ (Self-report Walking and Exercise Tables, 17 days)	-	▪ SSC ✓ ▪ ITT - ▪ CBD ✓ ▪ DOA ✓	✓	✓	-
Prochaska et al., 2008 [51]	4	RCT	IG1 (*n* = 433) ^c^ IG2 (*n* = 503) ICG (*n* = 464)	✓ (Self-reported level of exercise, 7 days)	-	▪ SSC ✓ ▪ ITT - ▪ CBD ✓ ▪ DOA -	✓	-	-
Sabti et al., 2010 [52]	2	Cohort study	IG (*n* = 776) Non-ICG (*n* = 463)	✓ (Evaluation questions based on HEPA survey, 7 days)	-	▪ SSC - ▪ ITT - ▪ CBD ✓ ▪ DOA -	-	-	-
Sherman et al., 2007 [53]	1	Cohort study	IG (*n* = 60) No CG	-	✓ (Pedometer, 3 days^a^)	▪ SSC - ▪ ITT - ▪ CBD - ▪ DOA -	-	-	-
Spittaels et al., 2007 [54]	6	Randomized clinical trial	IG1 (*n* = 116) IG2 (*n* = 122) ICG (*n* = 141)	✓ (IAPQ, 7 days)	-	▪ SSC - ▪ ITT ✓ ▪ CBD ✓ ▪ DOA ✓	-	-	-
Spittaels et al., 2007 [55]	5	RCT	IG1 (*n* = 173) IG2 (*n* = 129) Non-ICG (*n* = 132)	✓ (IPAQ, 7 days)	-	▪ SSC - ▪ ITT ✓ ▪ CBD ✓ ▪ DOA ✓	-	-	-
Steele, Mummery, & Dwyer, 2007 [56]	6	Randomized trial	IG1 (*n* = 65) IG2 (*n* = 65) IG3 (*n* = 62) No CG	✓ (Active Australian Questionnaire, 7 days)	-	▪ SSC ✓ ▪ ITT ✓ ▪ CBD ✓ ▪ DOA -	✓	✓	✓ (2 and 5 months, significant)
Sternfeld et al., 2009 [57]	6	RCT	IG (*n* = 351) Non-ICG (*n* = 436)	✓ (PAQ, adapted from Cross-Cultural Activity Patterns Questionnaire, 7 days in last 4 months)	-	▪ SSC - ▪ ITT ✓ ▪ CBD ✓ ▪ DOA -	-	-	✓ (4 months, significant)
Yap et al., 2009 [58]	0	Quasi-experimental design	IG (*n* = 37) Non-ICG (*n* = 36)	✓ (Stanford Brief Activity Survey, 2 weeks)	✓ (Accelerometer, 24 hours)	▪ SSC - ▪ ITT - ▪ CBD - ▪ DOA -	-	-	-
Zoellner et al., 2010 [59]	2	Feasibility study	IG (*n* = 56) No CG	-	✓ (Pedometer, 1 month ^a^)	▪ SSC - ▪ ITT - ▪ CBD - ▪ DOA -	-	-	-

✓: used/done; -: not used/not done/not reported; CBD: check for baseline-differences; CG: control group, whose participants received no intervention; DOA: drop-out analysis; ICG: control group that gets any form of intervention, including usual care and placebos; IG: intervention group; ITT: intention-to-treat analyses; PA: physical activity; RCT: randomized controlled trial; SSC: sample size calculation. ^a^ Recorded daily steps were reported in a step log. ^b^ *n* for at-risk subsample; total population for analysis IG *n* = 827, CG *n* = 890. ^c^ Sample size for the analysis not reported in the article; baseline values listed above.

## Supplementary Materials

The supplementary materials are available online at https://www.mdpi.com/1660-4601/16/5/813/s1.
