# Supplementary material for: Effectiveness Studies in Health Promotion: A Review of the Methodological Quality of Studies Reporting Significant Effects on Physical Activity in Working Age Adults
Source: Int J Environ Res Public Health. 2019 Mar 6;16(5):813. doi: 10.3390/ijerph16050813 (PMC6427597; doi:10.3390/ijerph16050813)
Supplement: Supplementary file 1 [file ijerph-16-00813-s001.pdf]

International Journal of
Environmental Research and Public Health

Supplementary Material

Effectiveness Studies in Health Promotion: A Review of the Methodological Quality of Studies Reporting Significant Effects on Physical Activity in Working Age Adults

Kevin Rudolf, Lea A. L. Dejonghe, Ingo Froböse, Florian Lammer, Lisa-Marie Rückel, Jessica Tetz and Andrea Schaller

References of reviews of primary studies which were included in the present review.

1. Bender, M.S.; Choi, J.; Won, G.Y.; Fukuoka, Y. Randomized controlled trial lifestyle interventions for Asian Americans: a systematic review. *Preventive medicine* **2014**, *67*, 171–181.

2. Blackman, K.C.; Zoellner, J.; Berrey, L.M.; Alexander, R.; Fanning, J.; Hill, J.L.; Estabrooks, P.A. Assessing the internal and external validity of mobile health physical activity promotion interventions: a systematic literature review using the RE-AIM framework. *Journal of medical Internet research* **2013**, *15*, e224.

3. Bock, C.; Jarczok, M.N.; Litaker, D. Community-based efforts to promote physical activity: a systematic review of interventions considering mode of delivery, study quality and population subgroups. *Journal of science and medicine in sport* **2014**, *17*, 276–282.

4. Broekhuizen, K.; Kroeze, W.; van Poppel, M.N.M.; Oenema, A.; Brug, J. A Systematic Review of Randomized Controlled Trials on the Effectiveness of Computer-Tailored Physical Activity and Dietary Behavior Promotion Programs: an Update. *Annals of Behavioral Medicine* **2012**, *44*, 259–286.

5. Buchholz, S.W.; Wilbur, J.; Ingram, D.; Fogg, L. Physical activity text messaging interventions in adults: a systematic review. *Worldviews on evidence-based nursing* **2013**, *10*, 163–173.

6. Cleland, V.; Granados, A.; Crawford, D.; Winzenberg, T.; Ball, K. Effectiveness of interventions to promote physical activity among socioeconomically disadvantaged women: a systematic review and meta-analysis. *Obesity reviews : an official journal of the International Association for the Study of Obesity* **2013**, *14*, 197–212.

7. Dugdill, L.; Brettle, A.; Hulme, C.; McCluskey, S.; Long, A.F. Workplace physical activity interventions: a systematic review. *Intl J of Workplace Health Mgt* **2008**, *1*, 20–40.

8. Gagliardi, A.R.; Abdallah, F.; Faulkner, G.; Ciliska, D.; Hicks, A. Factors contributing to the effectiveness of physical activity counselling in primary care: a realist systematic review. *Patient education and counseling* **2015**, *98*, 412–419.

9. Gourlan, M.J.; Trouilloud, D.O.; Sarrazin, P.G. Interventions promoting physical activity among obese populations: a meta-analysis considering global effect, long-term maintenance, physical activity indicators and dose characteristics. *Obesity reviews : an official journal of the International Association for the Study of Obesity* **2011**, *12*, e633-45.

10. Head, K.J.; Noar, S.M.; Iannarino, N.T.; Grant Harrington, N. Efficacy of text messaging-based interventions for health promotion: a meta-analysis. *Social science & medicine (1982)* **2013**, *97*, 41–48.

International Journal of
Environmental Research and Public Health

11. Kwak, L.; Hagströmer, M.; Jensen, I.; Karlsson, M.L.; Alipour, A.; Elinder, L.S. Promoting physical activity and healthy dietary behavior: the role of the occupational health services: a scoping review. *Journal of occupational and environmental medicine* **2014**, *56*, 35–46.

12. Lewis, Z.H.; Lyons, E.J.; Jarvis, J.M.; Baillargeon, J. Using an electronic activity monitor system as an intervention modality: A systematic review. *BMC public health* **2015**, *15*, 585.

13. Marcus, B.H.; Ciccolo, J.T.; Sciamanna, C.N. Using electronic/computer interventions to promote physical activity. *British journal of sports medicine* **2008**, *43*, 102–105.

14. Olander, E.K.; Fletcher, H.; Williams, S.; Atkinson, L.; Turner, A.; French, D.P. What are the most effective techniques in changing obese individuals' physical activity self-efficacy and behaviour: a systematic review and meta-analysis. *The international journal of behavioral nutrition and physical activity* **2013**, *10*, 29.

15. Richards, J.; Thorogood, M.; Hillsdon, M.; Foster, C. Face-to-face versus remote and web 2.0 interventions for promoting physical activity. *The Cochrane database of systematic reviews* **2013**, CD010393.

16. Short, C.E.; James, E.L.; Plotnikoff, R.C.; Girgis, A. Efficacy of tailored-print interventions to promote physical activity: a systematic review of randomised trials. *The international journal of behavioral nutrition and physical activity* **2011**, *8*, 113.

17. Spana, T.M.; Rodrigues, R.C.M.; Lourenço, L.B.d.A.; Della Mendez, R.R.; Gallani, M.C.B.J. Integrative review: behavioral interventions for physical activity practice. *Revista latino-americana de enfermagem* **2009**, *17*, 1057–1064.

18. Vuillemin, A.; Rostami, C.; Maes, L.; van Cauwenberghe, E.; van Lenthe, F.J.; Brug, J.; Bourdeaudhuij, I. de; Oppert, J.-M. Worksite physical activity interventions and obesity: a review of European studies (the HOPE project). *Obesity facts* **2011**, *4*, 479–488.

19. Whitt-Glover, M.C.; Keith, N.R.; Ceaser, T.G.; Virgil, K.; Ledford, L.; Hasson, R.E. A systematic review of physical activity interventions among African American adults: evidence from 2009 to 2013. *Obesity reviews : an official journal of the International Association for the Study of Obesity* **2014**, *15 Suppl 4*, 125–145.

20. Williams, G.; Hamm, M.P.; Shulhan, J.; Vandermeer, B.; Hartling, L. Social media interventions for diet and exercise behaviours: a systematic review and meta-analysis of randomised controlled trials. BMJ open 2014, 4, e003926.

© 2019 by the authors. Submitted for possible open access publication under the terms and conditions of the Creative Commons Attribution (CC BY) license (http://creativecommons.org/licenses/by/4.0/).
